# Construction and validation of a hypoxia-related risk signature identified EXO1 as a prognostic biomarker based on 12 genes in lung adenocarcinoma

**DOI:** 10.18632/aging.204613

**Published:** 2023-03-25

**Authors:** Qirui Chen, Shuo Chen, Jing Wang, Yan Zhao, Xin Ye, Yili Fu, Yi Liu

**Affiliations:** 1 Department of Thoracic Surgery, Beijing Chaoyang Hospital, Beijing 100020, China

**Keywords:** lung adenocarcinoma, hypoxia, bioinformatics analysis, TCGA

## Abstract

Background: Increasing evidence has demonstrated the clinical importance of hypoxia and its related factors in lung adenocarcinoma (LUAD).

Methods: RNA-seq datasets from The Cancer Genome Atlas (TCGA) were analyzed using the differentially expressed genes in hypoxia pathway by the Least Absolute Shrinkage and Selection Operator (LASSO) model. Applying gene ontology (GO) and gene set enrichment analysis (GSEA), a risk signature associated with the survival of LUAD patients was constructed between LUAD and normal tissue.

Results: In total, 166 hypoxia-related genes were identified. Based on the LASSO Cox regression, 12 genes were selected for the development of the risk signature. Then, we designed an OS-associated nomogram that included the risk score and clinical factors. The concordance index of the nomogram was 0.724. ROC curve showed better predictive ability using the nomogram (AUC = 0.811 for 5-year OS). Finally, the expressions of the 12 genes were validated in two external datasets and EXO1 was recognized as a potential biomarker in the progression of LUAD patients.

Conclusions: Overall, our data suggested that hypoxia is associated with the prognosis, and EXO1 acted as a promising biomarker in LUAD.

## INTRODUCTION

Lung adenocarcinoma (LUAD) is the most aggressive histological type of lung cancer with a consistently increasing incidence [1, 2]. The lack of early detection and effective treatment led to the persistently high mortality rate of LUAD, which makes it urgent to explore the mechanisms of LUAD. However, the traditional experimental approach limits the large-scale investigation of hub genes and pathways at the systems biology level because it can only identify one or a few genes at a time. Machine learning is a new approach to learn data-driven concepts which can help researchers uncover hidden insights [3], and it is also developed for precise classification and accurate prediction in medicine by designing models according to RNA-sequencing patterns. The molecular characteristics and therapy effects vary widely between different subtypes of lung cancer [4]. Several high-frequency genetic alternations have been identified in LUAD, such as LAMA2, DGCR5, and TTC21A [5–7].

The interaction between cancer cells and the tumor (specifically the hypoxia) microenvironment has been widely shown to affect the LAUD progression and therapy. Hypoxia can decrease the activity of immune cells and suppress the production of immune stimulants, thereby increasing the release of inhibitory factors and improving the expression of immune checkpoint inhibitors [8, 9]. Hypoxia, as a promising therapeutic target especially for radiotherapy, has been applied to most of LUAD patients. However, LUAD hypoxia-targeted therapy is still in clinical trials [10].

In this study, hypoxia-related genes (HRGs) with different expressions were screened by using large-scale sequencing and bioinformatics analysis. Hypoxia-related prognostic model was constructed via LASSO-Cox regression analysis. A prognostic nomogram, which combined prognostic gene characteristics and clinical prognostic factors, was created to predict the overall survival (OS). And the EXO1 expression levels in tumor and adjacent normal tissues were examined and the prognostic value of EXO1 in LUAD patients was evaluated.

## MATERIALS AND METHODS

### Data acquisition and procession

The expression profiles of mRNAs (level 3) in 504 cases (504 tumor tissues and 59 adjacent normal tissues) were downloaded from TCGA database (https://portal.gdc.cancer.gov). And its corresponding clinical information of LUAD patients was also downloaded from TCGA and was shown in Supplementary Table 1. Hypoxia-related genes (HRGs) were derived from the gene set in the Molecular Signatures Database (MSigDB v6.2, http://software.broadinstitute.org/gsea/msigdb) [11]. Transcriptome profiling data of LUAD patients in the GSE19188 (91 tumor- and 65 adjacent normal samples) and GSE10072 (58 tumor and 49 non-tumor tissues) datasets from the GEO database were used for validation.

### Identification of hypoxia-related genes and functional enrichment analysis

With the cut-off criteria set as log2|Fold Change| > 1 and *P*-value < 0.05, we normalized these data and screened differentially expressed hypoxia-related genes (DE-HRGs) using “limma” package by R software [12]. Gene ontology (GO) [13] and Kyoto Encyclopedia of Genes and Genomes (KEGG) [14] pathway enrichment analyses were conducted to explore the biological functions of the DE-HRGs via the “clusterProfiler” R package. Adjusted *P*-value < 0.05 was set as the significance threshold, and the enrichment analysis result maps were presented by the “ggplot2” and “GOplot” R packages.

### Construction and evaluation of the hypoxia-related prognostic model

We performed the least absolute shrinkage and selection operator (LASSO) regression analysis to select hub prognosis-related DE-HRGs via the “glmnet” R package. The formula of the risk score for the predicting patients’ survival was as follows:


$$\text{Risk score}=\sum {\text{n}}_{\text{i}}=\sum \left(\text{Coefi}\times {\text{x}}_{\text{i}}\right)$$

where Coefi refers to the coefficient and x_i_ refers to the expression level of HRGs. Based on the median value of risk scores, patients were divided into high-risk and low-risk subgroups, in which the clinic pathological features and gene expression profiles of each patient were displayed through the “pheatmap” and “survival” R packages. The OS rates between the two groups were compared by the Kaplan-Meier survival analysis in R package (*P* < 0.05). The predictive accuracy of the risk model was further verified by the receiver operating characteristic (ROC) curve.

### Gene set enrichment analysis (GSEA)

GSEA was conducted in the molecular signatures database (MSigDB), which provided hallmark gene sets to predict biological processes between low- and high-risk groups [15]. Annotated gene sets of hallmarker.all.v6.1.symbols.gmt in MSigDB were chosen as the references in GSEA software [16]. *P* < 0.05 and FDR < 0.25 was considered as obviously enriched.

### Estimation of clinical independence and construction of the nomogram

Univariate and multivariate Cox regression analyses were used to determine whether the prognostic model was an independent risk factor. We then used the independent risk factors to establish the nomogram and calibration curves by the “rms” package in R language. The accuracy was examined by checking the consistency index between actual and predicted probability. Next, we showed the predicted and observed results in the calibration curve to visualize the performance of the nomogram, and the 45° line represented the best prediction. The prognostic evaluation of nomogram was then performed with Kaplan–Meier survival analysis and the area under the tdROC curve (AUC). Finally, we measured Harrel's concordance index (C-index) to validate the predictive capability of the nomogram [17].

### *In vitro* validation of hub genes

Proteins were extracted and western blot was performed as previous described [18]. Total RNA was extracted using TRIzol reagent (Tiangen, China). cDNAs were reverse transcribed into mRNA through PrimeScript RT-polymerase (Vazyme). qRT-PCR was carried out by using SYBR-Green Premix (Vazyme) with specific PCR primers (ThermoFisher, USA), with Glyceraldehyde-3-phosphate dehydrogenase as an internal reference. The gene expression level was analyzed using the 2^−ΔΔCt^ method. All steps of qRT-PCR follow the instructions.

## RESULTS

### Differentially expressed HRGs in patients with LUAD

**Figure 1:**
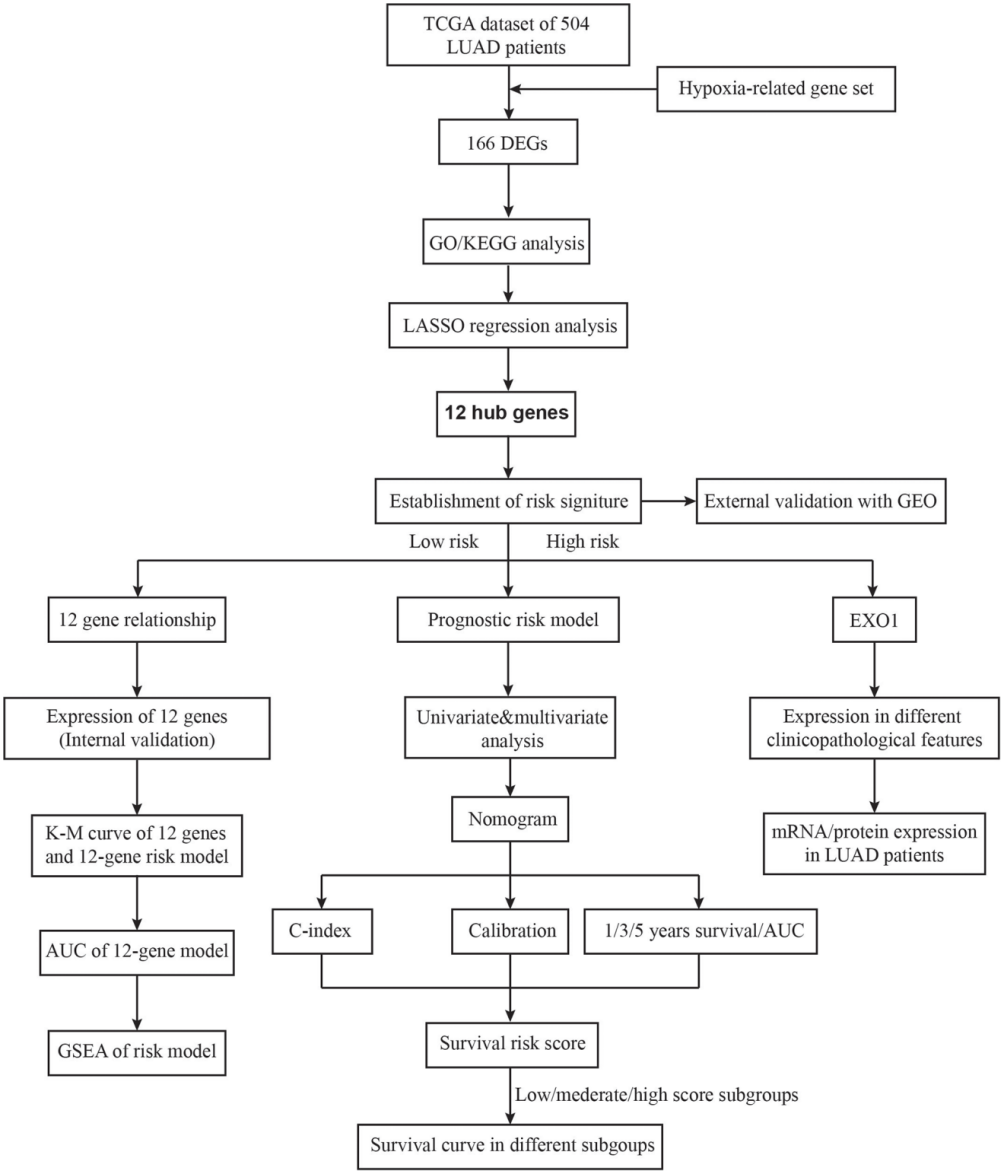
The flow chart of the study design.

**Figure 2:**
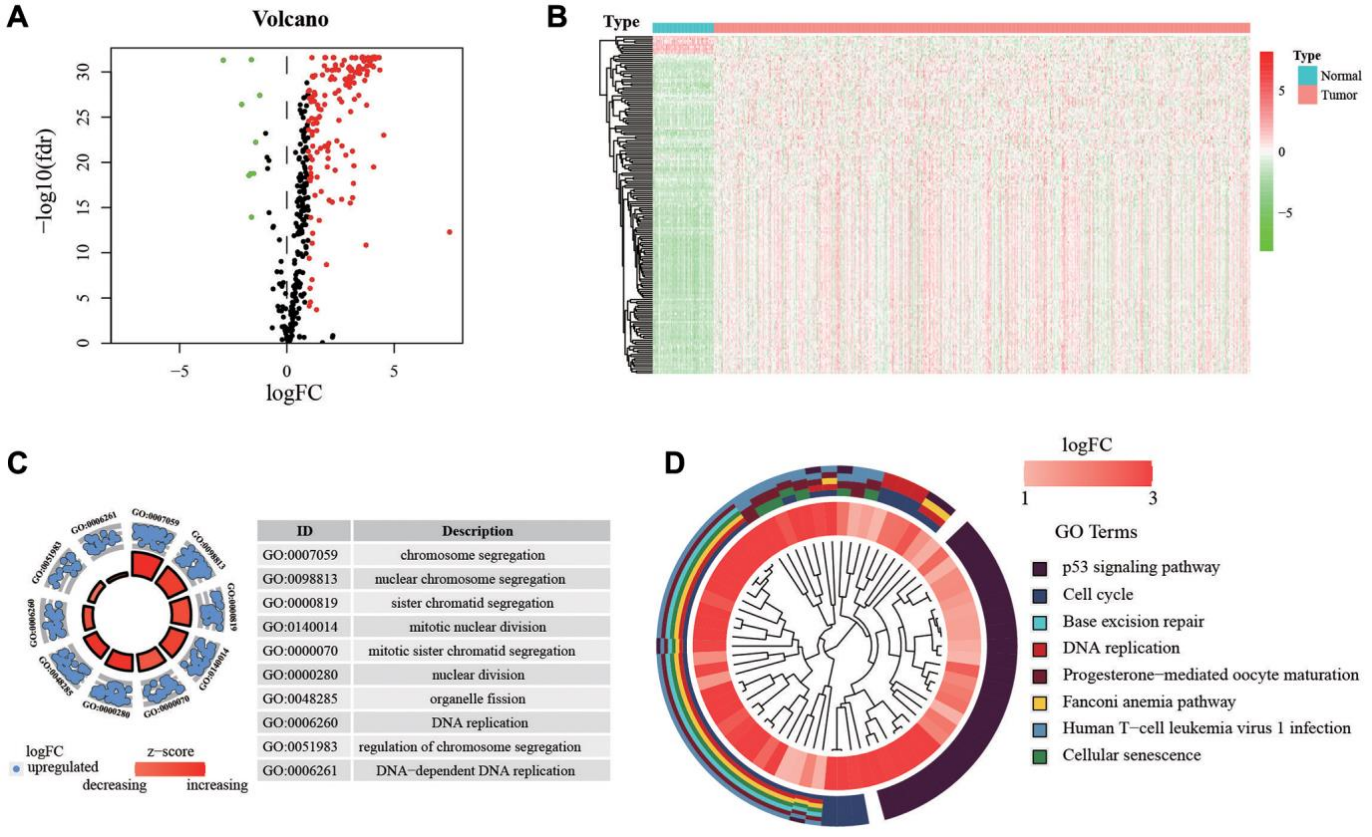
**Expression of genes and function enrichment.** (**A**) Volcano plot and (**B**) heatmap showing the DEGs between LUAD and normal lung samples. Red dots represent up-regulated and green dots represent down-regulated DEGs, black dots represent no difference, respectively (log FC >1, *P* < 0.05). (**C**) GO analysis showing the differentially expressed hypoxia-related genes. (**D**) The significantly enriched pathways of the DEGs determined by KEGG analysis. Abbreviations: GO: gene ontology; KEGG: Kyoto Encyclopedia of Genes and Genomes.

The design of the study was shown in Figure 1. First, we extracted the expression of HRGs from the TCGA database. These HRGs were performed with differentially expressed analysis, and 166 differentially expressed HRGs were obtained, including 9 down-regulated and 157 up-regulated genes (Figure 2A, 2B). During enrichment analyses for these DE-HRGs, these genes were mainly found to play a role in “chromosome segregation”, “nuclear chromosome segregation”, “sister chromatid segregation”, and “mitotic nuclear division” for GO enrichment, and “Cell cycle”, “DNA replication”, and “p53 signaling pathway” as shown in Kyoto Encyclopedia of Genes and Genomes (KEGG, Figure 2C, 2D). The above findings suggested that these DE-HRGs were strongly associated with the development, progression and proliferation of LUAD cells.

### Establishment of a 12 HRGs-based prognostic signature

**Figure 3:**
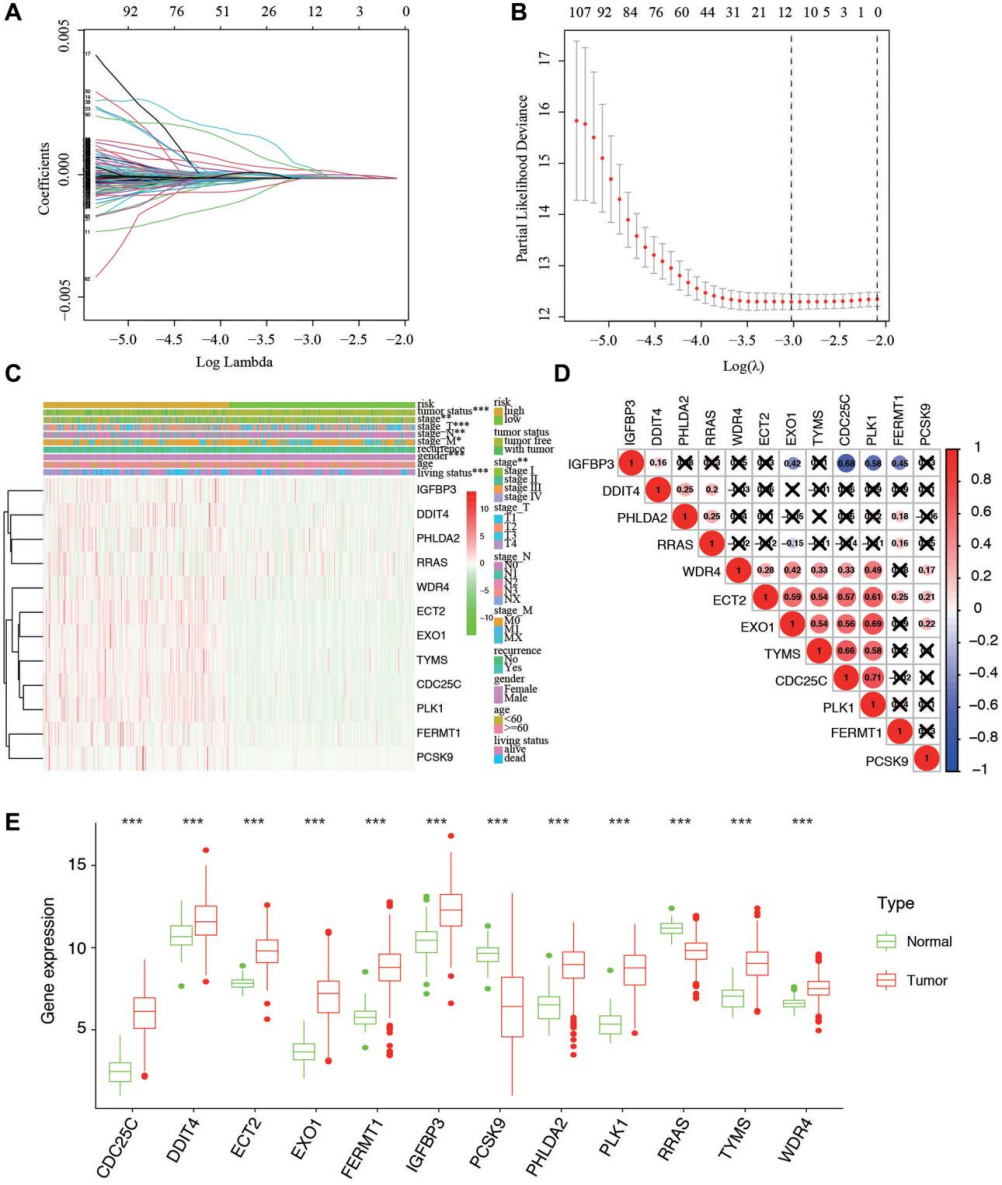
**Ten-fold cross-validation for tuning parameter selection and a gene expression.** (**A**) Plots of the ten-fold cross-validation error rates. (**B**) LASSO coefficient profiles of the twelve hypoxia-related genes. (**C**) Relationship between the risk score and clinical significance. (^***^P-value < 0.001, ^**^P-value < 0.01, and ^*^P-value < 0.05). (**D**) Associations between the 12 genes. (**E**) The expression of the 12 genes in different types of tissues.

For screening out the DE-HRGs with potentially prognostic value for LUAD patients, LASSO regression was used. LASSO analyses identified 12 genes significantly associated with prognosis: IGFBP3, DDIT4, PHLDA2, RRAS, WDR4, EXO1, ECT2, TYMS, CDC25C, PLK1, FERMT1, and PCSK9. (Figure 3A, 3B). According to the results of LASSO regression analysis, we used the coefficients (Table 1) to construct the prognostic model as following: risk score = (CDC25C × 0.37358) + (DDIT4 × 0.34287) + (ECT2 × 0.12761) + (EXO1 × 0.29731) + (FERMT1 × 0.89012) + (IGFBP3 × 0.29168) + (PCSK9 × 0.52722) + (PHLDA2 × 0.40304) + (PLK1 × 0.91384) + (RRAS × 0.43750) + (TYMS × 0.49112) + (WDR4 × 0.26711). Patients were ranked in ascending order by score and divided into high- and low-risk subgroups using median risk values as a reference. Besides, the expression of the 12 genes in low- and high-risk patients in the TCGA dataset was also demonstrated in the heatmap. The expression profiles of the 12 prognostic DE-HRGs showed that all of the 12 genes were expressed at higher levels in the high-risk subgroup. We also found significant differences between the high- and low-risk groups associated with tumor status, stage, stage_T, stage_N, stage_M, gender, and living status (Figure 3C). We further analyzed the relationship between the 12 genes. We found that they were significantly relevant, especially between EXO1 and PLK1, ECT2 and PLK1, IGFBP3 and CDC25C, TYMS and CDC25C (Figure 3D). Furthermore, we analyzed the gene expression of the 12 genes in different tissues types. We found that expression levels of PCSK9 and RRAS were significantly higher in normal tissue, and the rest 10 genes were overexpressed in tumor tissues (Figure 3E).

**Table 1:** Twelve hypoxia-associated genes and corresponding coefficient value.

**Hypoxia associated gene**	**Coefficient**
IGFBP3	0. 29168
DDIT4	0. 34287
PHLDA2	0. 40304
RRAS	0. 43750
WDR4	0. 26711
EXO1	0. 29731
ECT2	0. 12761
TYMS	0. 49112
CDC25C	0.37358
PLK1	0. 91384
FERMT1	0. 89012
PCSK9	0. 52722
Risk score	Low: < 1.66
	High: ≥ 1.66

### Efficiency of predicting the patients' outcomes with the 12 HRGs signature

**Figure 4:**
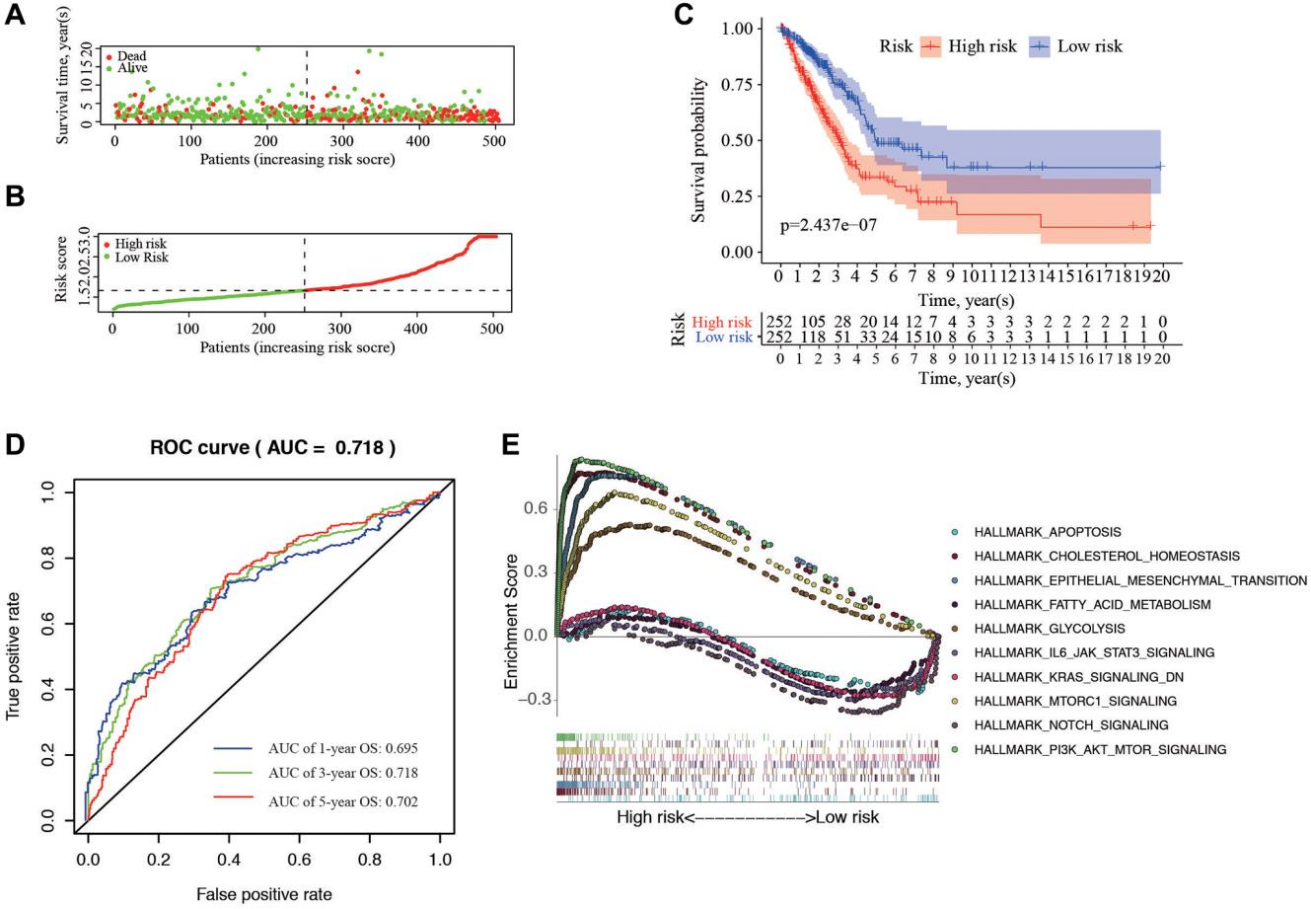
**Correlation between the risk score and overall survival.** (**A**, **B**) Distribution of risk score and patient survival status of LUAD. (**C**) The Kaplan–Meier curve demonstrates that patients in the high-risk group have a poorer prognosis. (**D**) Time-dependent ROC curve of 1-, 3-, and 5-year analysis for survival prediction by the risk score. (**E**) Hallmark analysis of Gene Set Enrichment Analysis (GSEA) in high-risk and low-risk groups.

The individual risk score and survival status was shown on the dot plot, with significantly different OS between two groups (Figure 4A, 4B). As indicated by Kaplan-Meier curve, the higher-risk group has significantly shorter OS than the low-risk group (*P* = 2.437e−07) (Figure 4C). The area under the ROC curve (AUC) of the prognostic HRGs model for 1-, 3-, 5-year OS was 0.695, 0.718, and 0.702, respectively (Figure 4D). GSEA analysis showed that the high-risk samples were mainly enriched in pathways of apoptosis, cholesterol homeostasis, and EMT; while the low-risk samples were mainly in PI3K-AKT-mTOR, Notch, and mTORC1 signaling pathways (Figure 4E).

### Construction and validation of nomogram for OS prediction

**Figure 5:**
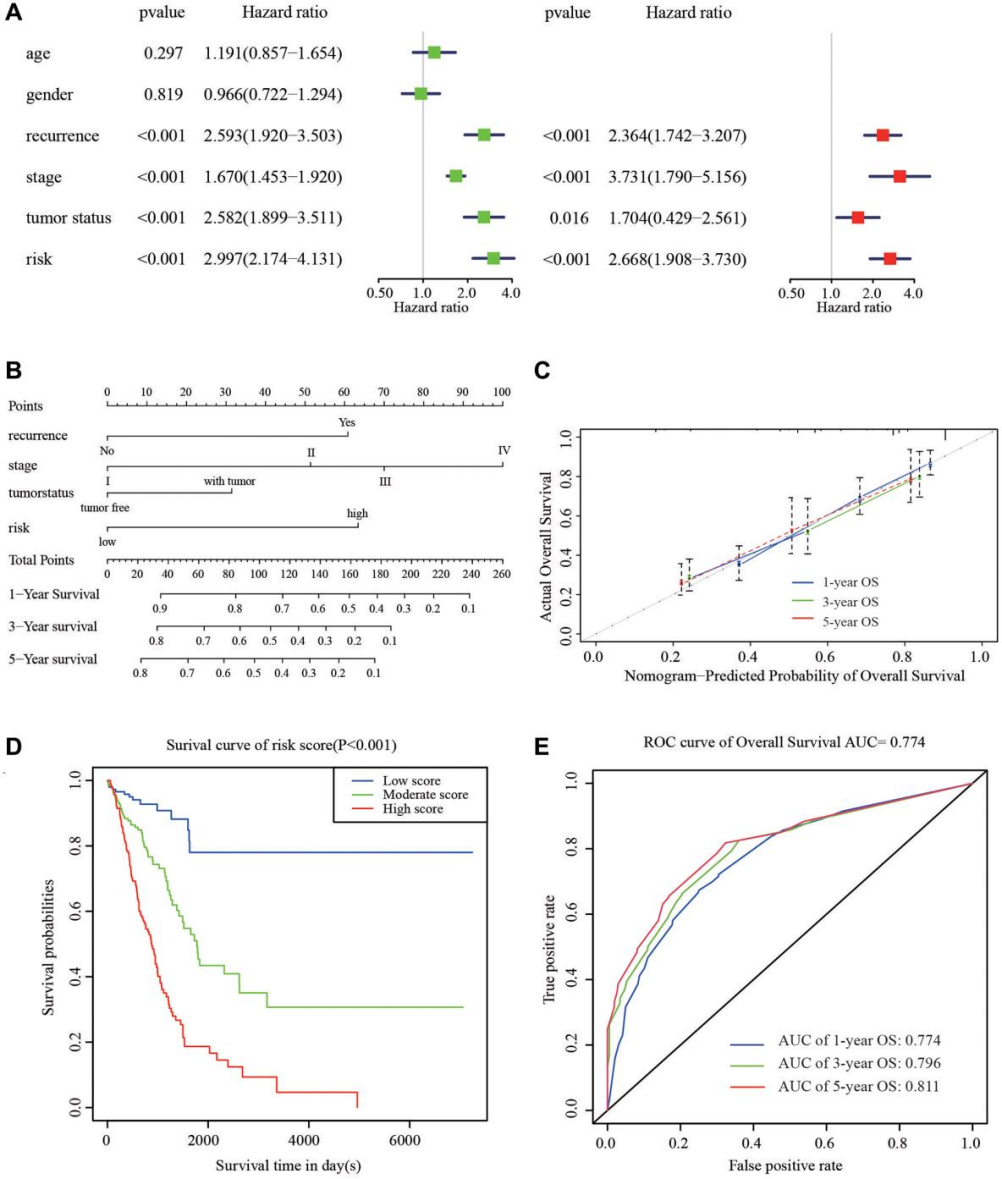
**Nomogram to predict the probability of patients with LUAD.** (**A**) Univariate and multivariate regression analyses of the prognostic value of clinicopathological features. (**B**) The nomogram to predict 1-, 3-, or 5-year OS in the LUAD patients. (**C**) The calibration plots for predicting patient 1-, 3-, or 5-year OS. (**D**) The Kaplan–Meier curves represent the survival probability of low, moderate, and high score group patients based on the nomogram. (**E**) The ROC curves of the nomogram of 1-, 3-, and 5-year survival.

To supply a better quantitative approach to predict the prognosis of cancer patients, a prognostic nomogram was built by integrating the all independent OS risk score and other clinicopathological risk factors. Cox regression analysis (Univariate and multivariate) was performed using clinicopathological and HRGs features for the OS of LUAD prediction. Recurrence, stage, tumor status, and risk signature were all taken as the independent risk factors of OS (Figure 5A), and they were used to establish OS nomogram (Figure 5B). The predicted value was consistent with the actual 1-, 3-, and 5-year OS time, as displayed the nomogram (Figure 5C). The score details of different factors were shown in Table 2. The C-index of the model was 0.75 (95% CI: 0.68–0.82). Based on the risk scores from the nomogram, the cohort was evenly divided into 3 subgroups (low-, moderate-e, and high-score groups), in which the high-score group had a worse OS than the moderate- and low-score groups (Figure 5D). The AUC of 1-, 3-, 5-year OS was 0.774, 0.796, and 0.811, respectively, which represent the predictive abilities of the nomogram (Figure 5E), suggesting that the nomogram had a high accuracy in predicting OS.

**Table 2:** Corresponding risk score for each variable and total score.

**Variables**	**Category**	**Score**
Recurrence	Recurrence	0
	Recurrence free	60
Stage	I	0
	II	50
	III	70
	IV	100
Tumor status	Tumor free	0
	With tumor	35
Risk signature	Low	0
	High	75
Total score	Low risk	0–50
	Moderate risk	60–125
	High risk	≥130

### Verification of 12 genes for prognostic prediction

**Figure 6:**
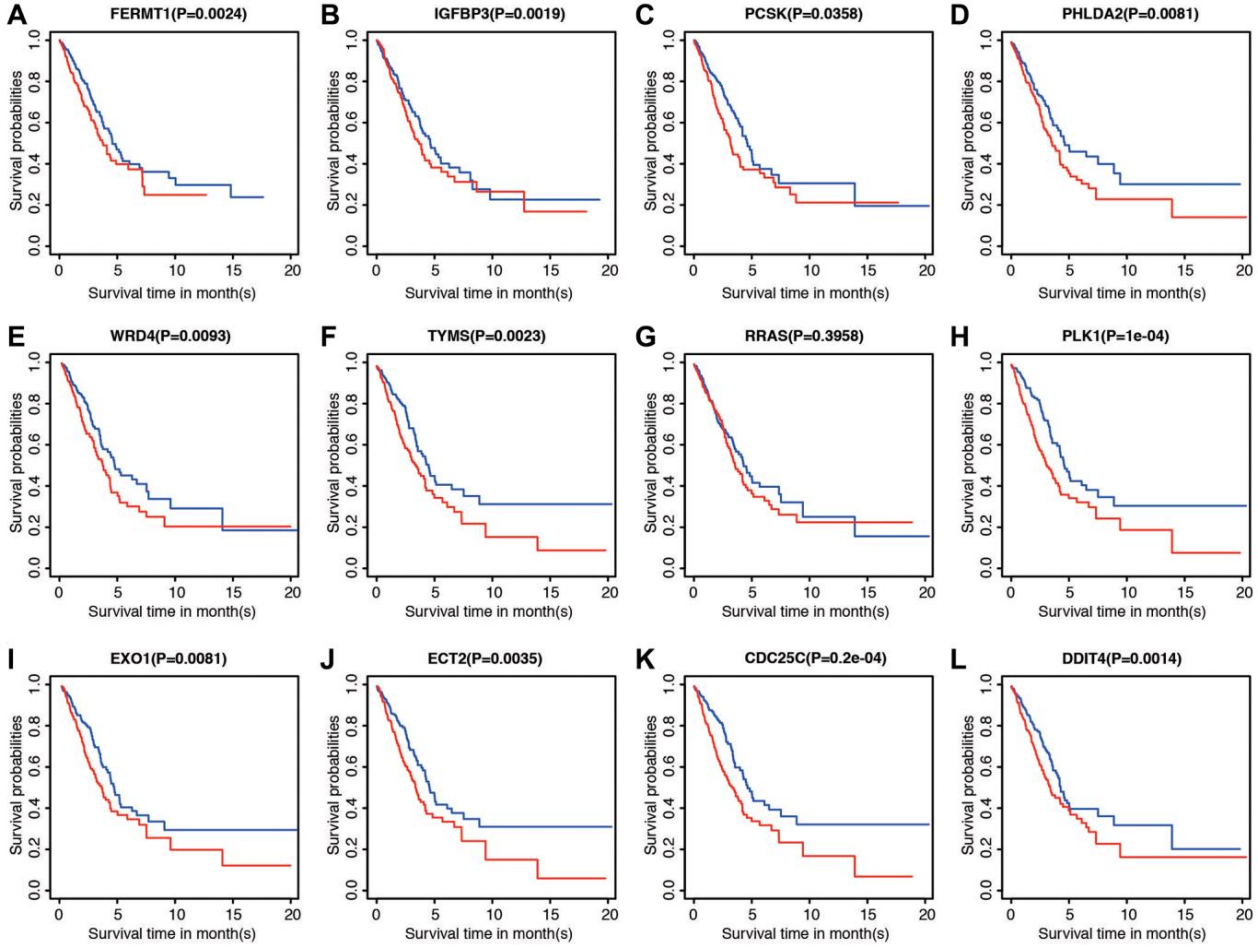
**Prognostic significance of low and high expression of each of the 12 genes.** (**A**) FERMT1. (**B**) IGFBP3. (**C**) PCSK. (**D**) PHLDA2. (**E**) WRD4. (**F**) TYMS. (**G**) RRAS. (**H**) PLK1. (**I**) EXO1. (**J**) ECT2. (**K**) CDC25C. (**L**) DDIT4.

The correlations between each gene from the prognostic model and the patients’ clinical OS were also measured. Patients were divided into two cohorts according to median value of each gene. The results indicated that eleven of the twelve genes, except RRAS, were shown to be significantly associated with patient prognosis (Figure 6A–6L).

### Expression of the 12 genes in database and *in vitro*

**Figure 7:**
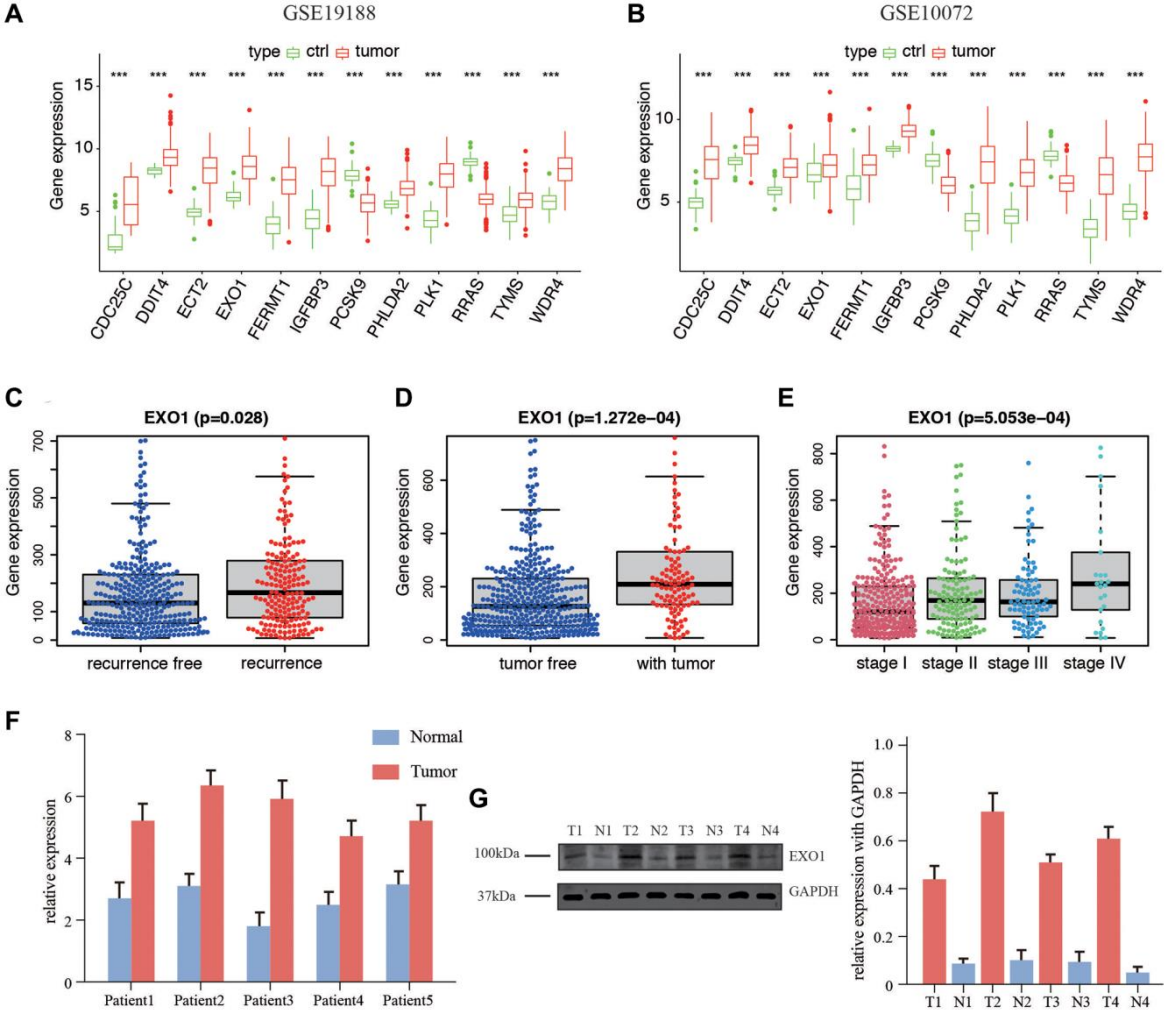
**Expression of the 12 genes in database and *in vitro* validation of EXO1.** (**A**) Gene expression in GSE19188 and (**B**) GSE10072. Expression of EXO1 in different (**C**) recurrence subgroups, (**D**) tumor status, and (**E**) stages. (**F**) Relative expression of EXO1 in tumor and normal tissues of 5 patients in our center for RNA level. (**G**) Protein level of EXO1 in patients in our center, and barplot shows its relative expression. N represents normal tissue, and T for tumor tissues.

We validated the expression of 12 genes in two GEO datasets, including GSE19188 and GSE 10072, by comparing the content between normal tissues and tumor tissues. The results were corresponding with that in TCGA datasets. Only PCSK9 and RRAS were overexpressed in normal tissues, and the rest of ten genes were higher in tumor tissues (Figure 7A, 7B). We then examined the correlation between the EXO1 and clinicopathological characteristics of the LUAD patients in TCGA. The analysis showed that EXO1 was statistically different in worse outcomes such as recurrence (Figure 7C), living with tumor (Figure 7D), and higher stage (Figure 7E). We performed the qPCR and western blot (WB) validation in clinical specimens following the steps described above. We verified the expression of EXO1 in the LUAD tissues and their adjacent normal tissues. By analysis, the qPCR results showed EXO1 was significantly up-regulated in the LUAD tissues, while down-regulated in normal LUAD tissues (Figure 7F). Results of WB indicated similar phenomenon (Figure 7G). These results suggested that EXO1 may play a pivotal role in the progression of LUAD.

## DISCUSSION

LUAD has become a major global health concern and the leading cause of cancer-associated mortality [19]. Although there has been more information about LUAD prognostic and predictive signatures, developing individualized treatments and prediction outcome is still challenging. Hypoxia is a common feature of most solid tumors, and it has widespread effects on metabolism, angiogenesis, and metastasis [20]. hypoxia induces a HIF1A-dependent complex molecular response [21]. Crosstalk between hypoxia and cancer-related hallmarks/pathways causes hypoxia-derived aggressive phenotypes and therapeutic resistance, which can be used as biomarkers to predict the clinical outcomes of LUAD patients at stage I [22]. Since hypoxia is a crucial factor in cancer progression, it is necessary looking for hypoxia-related biomarkers to predict the prognosis and promote the immune therapy of LUAD patients [23]. As more information about genomic changes of LUAD becomes available, some prognostic and predictive signatures were discovered and this is helpful for personalized therapy. In this study, a novel 12-gene prognostic signature was validated, which can be used to identify high-risk LUAD patients with poor prognosis. What’s more, the content of these 12 genes was also validated with two GEO datasets. Further study indicated that EXO1 is significantly up-regulated in worse clinicopathological features including recurrence, living with tumor, and higher stage. We also validated the expression of EXO1 with our own samples in RNA and protein levels.

Previous studies have identified many molecular signatures that classify patients into different prognostic groups. Cheng identified a prognostic gene signature associated with microenvironment in LAUD, and the 3-year area under the ROC curves (AUC) of the risk model reached to 0.738 [24]. Another study established an immune-related signature that could predict prognosis and reflect the tumor immune microenvironment of LUAD patients. The risk model promoted individualized treatment and provided potential novel targets for immunotherapy of LUAD patients [25]. Similarly, we also built the predictor based on 12 HRGs and the model performed well with much more higher AUC of ROC than other studies [26, 27].

Hypoxia is often caused by a supply-demand imbalance of nutrient in the tumor microenvironment. Although the incidence and severity of hypoxia in a given patient population is variable, it is a feature of the physiology of most tumors and is particularly involved in mechanisms related to certain malignant features (e.g., metastasis and invasion) [28–30]. Hypoxia-related genes are usually involved in the corresponding pathways or regulating apoptosis of these processes [31]. Taken together, tumor hypoxia might be taken as the best target. Extensive reports have demonstrated that the hypoxic microenvironment is a main factor regulating the progression and metastasis of solid tumors (including lung cancer) [32, 33]. Under hypoxic conditions, cancer cells secrete angiogenic factors to promote the abnormal angiogenesis. In addition, hypoxia causes cancer cells to be insensitive to chemotherapy or radiotherapy by increasing the malignancy of the tumor and the ability of invasion and metastasis of cancer cells [34]. Thus, the hypoxic microenvironment is an important factor for patients with LUAD. Hypoxia-inducible factor 1 (HIF-1) is a major regulator of the hypoxic response, its function has been well characterized [35]. YTHDF1, an m^6^A-modified mRNA-binding protein, is a high-altitude adaptation gene that was positively selected during evolution and participates in both hypoxic adaptation and the pathogenesis of LUAD [36].

In the correlative analysis, EXO1 is found to be associated with TYMS, CDC25C, and PLK1, which is the most correlative genes. What’s more, the logFC of EXO1 is 3.9, ranking the highest in cancer progression genes. Therefore we take EXO1 as our target. The function of EXO1 is mainly involved in mismatch repair and recombination. In our study, we identified EXO1 played a key role in the progression of LUAD patients. EXO1 was involved in tumor mutational burden (TMB) and its clinical significance in prostate cancer [37]. Elevated expression of EXO1 is associated with carcinogenesis and poor prognosis in breast cancer, and might act as a biomarker for breast cancer treatment [38]. EXO1 inhibited the activity of cancer progression through PARP pathway. It acted as a novel therapeutic target that serves important roles in DNA damage response. A recent study investigating the prognostic value of TP53-associated immune genes in hepatocellular cancer identified and validated a two-gene (TREM1 and EXO1) prognostic model [39].

Exonuclease 1 (EXO1) interacted with mutator S homolog 2 (MSH2) to regulate the mismatch repair and recombination [40]. The EXO1expression was up-regulated in tumor tissue [41]. It was reported that EXO1 works as a guardian of our genome to reduce cancer progression by inducing DNA damage checkpoints and DNA damage repair [42]. EXO1 is also reported as a potential prognostic biomarker for LUAD, and it is related to the infiltrating levels of immune cells. In this study, EXO1 has higher expression level in LUAD than that in the para-cancerous tissues from public databases (*p* < 0.01). Survival analysis demonstrated the correlation between high EXO1 expression with poor LUAD prognosis (*p* < 0.01). Additionally, *in vitro* results showed that downregulation of EXO1 inhibited the migratory ability of lung cancer cells.

To further understand the molecular function and pathway of these HRGs and risk model, we conducted functional enrichment analysis of GO, KEGG pathways, and GSEA. By performing KEGG enrichment analysis, these hypoxia-related genes were enriched in several pathways in cancers, including “p53 signaling pathway”, “cell cycle”, and “DNA replication”. Similarly, according to GO analysis, the DEGs also mainly clustered in “DNA replication” and other cell cycle-related function. In recent years, knowledge of the hypoxia features of LUAD has increased, and the development of effective immunotherapeutic strategies for LUAD has attracted much attention. The role of hypoxia functions by regulating the tumor microenvironment in LUAD therapy, especially through expression and activity of cell cycle related proteins [43, 44].

GSEA revealed that the risk model was differentially enriched in apoptosis, epithelial mesenchymal transition, fatty acid metabolism, glycolysis, Notch signaling pathway, and PI3K-AKT-mTOR signaling pathway. The activation of the Notch and PI3K-AKT-mTOR signaling pathway is a critical event which frequently occur during LUAD development, and these signaling pathways are uncovered clinically relevant to hypoxia, making it a therapeutic target for the therapy of LUAD [45, 46]. The fatty acid metabolism is important for cell growth and apoptotic regulation, making it also a potential molecular target for cancer treatment and prevention [47, 48]. Our findings suggested that these 12-gene signature may be involved in the oncogenesis and progression of LUAD.

Our study has some limitations. First, analysis on the transcriptional level can reflect some aspects of patients’ status, but not global changes. Moreover, another independent cohort and *in vitro* or *in vivo* functional studies should be performed to validate our results. Finally, mechanism validation and external validation of the patients are in need for the risk model in our study.

## CONCLUSION

In general, we present and validate a robust prognostic model aggregating 12 signature genes based on hypoxia pathway that can be used to efficiently predict LUAD patient prognosis. However, the clinical role as well as the biological function of these 12 mRNAs needs to be further verified with more experiments, especially for EXO1, which had potential to become a therapeutic target for LUAD patients.

## Supplementary Materials

Supplementary Table 1
