# Supplementary material for: Construction and validation of a hypoxia-related risk signature identified EXO1 as a prognostic biomarker based on 12 genes in lung adenocarcinoma
Source: Aging (Albany NY). 2023 Mar 25;15(6):2293–307. doi: 10.18632/aging.204613 (PMC10085621; doi:10.18632/aging.204613)
Supplement: Supplementary Table 1 [file aging-15-204613-s001.pdf]

SUPPLEMENTARY TABLE

Supplementary Table 1. Characteristics of patients in TCGA LUAD dataset.

Variables	Whole cohort
Total number	504
Age (year)	65.80 ± 13.5
OS (day)	913.6 ± 729.3
Living status	
Alive	322 (63.89%)
Death	182 (36.11%)
Gender	
Male	233 (46.23%)
Female	271 (53.77%)
Stage_T	
T1	327 (64.88%)
T2	96 (19.05%)
T3	69(13.69%)
TX	12 (2.38%)
Stage_M	
M0	338 (67.06%)
M1	25 (4.96%)
MX	141 (27.98%)
Stage_N	
N0	327 (64.88%)
N1	96 (19.05%)
N2	71 (14.09%)
NX	10 (1.98%)
Stage	
Stage I	274 (54.36%)
Stage II	122 (24.20%)
Stage III	83 (16.47%)
Stage IV	25 (4.97%)
Recurrence	
No	320 (63.49%)
Yes	184 (36.51%)
Tumor status	
Tumor free	392 (77.78%)
With tumor	112 (22.22%)

Abbreviations: TCGA: The Cancer Genome Atlas; LUAD: Lung adenocarcinoma.
